# Dietary seaweed-derived polysaccharides improve growth performance of weaned pigs through maintaining intestinal barrier function and modulating gut microbial populations

**DOI:** 10.1186/s40104-021-00552-8

**Published:** 2021-03-10

**Authors:** Tiande Zou, Jin Yang, Xiaobo Guo, Qin He, Zirui Wang, Jinming You

**Affiliations:** 1 Jiangxi Province Key Laboratory of Animal Nutrition, College of Animal Science and Technology, Jiangxi Agricultural University, Nanchang, 330045 Jiangxi China; 2 Jiangxi Province Key Innovation Center for Industry-Education Integration of High-Quality and Safety Livestock Production, Nanchang, 330045 China

**Keywords:** Intestinal health, Microflora, Pigs, Seaweed-derived polysaccharides

## Abstract

**Background:**

Seaweed-derived polysaccharides (SDP) represent an attractive source of prebiotic nutraceuticals for the food and animal husbandry industry. However, the mechanism by which SDP from *Enteromorpha* mediates pig growth are not fully understood. This study aimed to investigate how SDP supplementation influences the growth performance and intestinal health in weaned pigs.

**Results:**

In Exp. 1, 240 weaned pigs were randomly assigned to four dietary treatments and fed with a basal diet or a basal diet containing 200, 400 or 800 mg/kg SDP, respectively, in a 21-day trial. Pigs on the 400 or 800 mg/kg SDP-supplemented group had greater ADG and lower F/G ratio than those on the control group (*P*<0.05). In Exp. 2, 20 male weaned pigs were randomly assigned to two treatments and fed with a basal diet (CON group) or a basal diet supplemented with 400 mg/kg SDP (the optimum does from Exp. 1), in a 21-day trial. Pigs fed the SDP diet had greater ADG, the concentrations of serum IL-6 and TNF-α and the activities of glutathione peroxidase, superoxide dismutase and catalase (*P*<0.05), and lower F/G, diarrhea rate, as well as serum *D*-lactate concentrations and diamine oxidase activity (*P*<0.05). Moreover, dietary SDP supplementation enhanced secretory immunoglobulin A content, villus height and villous height: crypt depth ratio in small intestine, as well as the lactase and maltase activities in jejunum mucosa (*P*<0.05). SDP supplementation elevated the mRNA levels of inflammatory response-related genes (*IL-6*, *TNF-α*, *TLR4*, *TLR6* and *MyD88*), and the mRNA and protein levels of ZO-1, claudin-1 and occludin in jejunum mucosa (*P*<0.05). Importantly, SDP not only increased the *Lactobacillus* population but also reduced the *Escherichia coli* population in cecum (*P*<0.05). Furthermore, SDP increased acetic acid and butyric acid concentrations in cecum (*P*<0.05).

**Conclusions:**

These results not only suggest a beneficial effect of SDP on growth performance and intestinal barrier functions, but also offer potential mechanisms behind SDP-facilitated intestinal health in weaned pigs.

## Introduction

The intestinal epithelial barrier, mainly involves a single layer of epithelial cells that are interconnected by tight junctions, plays a pivotal role in controlling absorption of nutrients and preventing pathogens and toxins from entering the systemic circulation [1, 2]. For neonatal mammals, the nutritional, physiological, and social stresses usually induce intestinal barrier disruption or enteropathy, which is accompanied by growth retardation and increasing the risk of diarrhea incidence [3–5]. Thus, developing a novel feeding strategy for improving intestinal epithelial barrier functions at early postnatal life stages has attracted considerable research interests worldwide.

Seaweed-derived polysaccharides (SDP) are one of the most abundant polysaccharides in marine organisms, which have been confirmed to exhibit wide variety of biological and pharmacological activities, including antioxidation [6], immunomodulation [7], anticoagulant activity [8] and gastrointestinal protection [9]. Collectively, these properties seem to impel SDP to serve as an effective dietary supplement for improving the growth performance and health of neonatal mammals. It has been shown that the benefits of seaweed polysaccharides (laminarin or fucoidan) as functional ingredients during the early stage may result from reduced intestinal inflammatory response and improved intestinal morphology [7, 10, 11]. Furthermore, *Laminaria* spp.-derived seaweed extract containing bioactive polysaccharides enhances growth performance and improves gut health in weaned pigs partly through the alteration of intestinal microbial environment and improvement of nutrient digestibility [12–14]. *Enteromorpha* is a kind of widespread green seaweed, which has been developed as a pharmaceutical product and healthcare food source [15]. Recent studies provide the new insights into the role of marine-derived polysaccharides from seaweed *Enteromorpha* in improving the gut health and antioxidant capacity in juvenile shrimps [16] and laying hens [6], respectively. However, to the best of our knowledge, the influence of SDP from *Enteromorpha* on health status and intestinal barrier functions in weaned pigs has not been investigated, and the underlying mechanisms still remain unclear.

Therefore, we hypothesize that SDP from *Enteromorpha* could improve the growth performance of weaned pigs, and the beneficial effects were related to the modulation of intestinal barrier integrity and function, immune response and changes of gut microbial fermentation. Specially, our objectives are to explain the underlying mechanism of SDP from *Enteromorpha* for improving intestinal health.

## Materials and methods

All experimental procedures used in this study were approved by the Animal Care and Use Committee of Jiangxi Agricultural University (permit No. JXAULL-20190098).

### Exp. 1

#### Animals, diets and experimental design

Two hundred and forty pigs (Duroc × Landrace × Yorkshire), weaned at 24 days with an initial average body weight (BW) of 6.79 ± 0.04 kg, were randomly assigned into one of four dietary treatments with six replicate pens per treatment (five barrows and five gilts per pen) on the basis of initial BW, sex and litter. The dietary treatments consisted of the basal diet (control group, CON) and basal diet supplemented with 200, 400 or 800 mg/kg SDP (provided by Qingdao Haida Biotechnology Co., Ltd., Qingdao, China). The SDP were produced from the seaweed *Enteromorpha prolifera*, which contains 4.82% protein, 2.9% moisture, 17487.6 mg/kg calcium, 15362.7 mg/kg magnesium, 24262.4 mg/kg potassium, 3.93 mg/kg arsenium, 1.22 mg/kg plumbum, and 0.86 mg/kg cadmium. The content of polysaccharides is more than 48%. According to the analysis of polysaccharide composition by high performance liquid chromatography (HPLC), the SDP consisted of five monosaccharides including rhamnose (Rha), glucose (Glc), glucuronic acid (GlcA), xylose (Xyl) and galactose (Gal). The molar percentage of monosaccharides is Rha: 40.6%, Glc: 38.2%, GlcA: 9.3%, Xyl: 6.3%, Gal: 5.6%. The basal diet was formulated to meet the National Research Council (2012)-recommended nutrient requirements for pigs at corresponding growth period, and the ingredient and nutrient composition were shown in Table 1. Pigs were fed isonitrogenous diets containing various levels of SDP. Although the additional SDP was added to the basal diet by substituting for the same amount of corn, the protein level is still low, which is basically consistent with protein in basal diet. All pigs were housed in a temperature-controlled room and had free access to water and feed throughout the trial. The experimental period lasted 21 days.

**Table 1:** Ingredients and nutrient composition of the basal diet for piglets (as-fed basis) (Exp. 1 and Exp. 2)

Ingredients	%	Nutrient composition	
Extruded corn	48.50	Calculated composition	
Soybean meal	11.00	Digestible energy, MJ/kg	14.82
Extruded soybean	10.00	Crude protein, %	19.64
Broken rice	9.00	Calcium, %	0.81
Flour	8.00	Total phosphorus, %	0.60
Whey powder	6.00	Available phosphorus, %	0.41
Soybean oil	1.05	Lysine, %	1.43
Fish meal	2.50	Methionine, %	0.54
Limestone	1.00	Methionine and cystine, %	0.83
Dicalcium phosphate	0.90	Threonine, %	0.98
NaCl	0.30	Tryptophan, %	0.20
*L*-Lysine HCl, 78%	0.60	Analysed composition	
*DL*-Methionine, 99%	0.26	Crude protein, %	18.69
*L*-Threonine, 98.5%	0.34	Calcium, %	0.78
Choline chloride	0.10	Total phosphorus, %	0.61
Vitamin premix^a^	0.05		
Mineral premix^b^	0.40		
Total	100		

^a^ Provided the following per kilogram of diet: vitamin A, 6000 IU; vitamin D_3_, 480 IU; vitamin E, 40 IU; vitamin K_3_, 1.5 mg; thiamin, 3 mg; riboflavin, 6.4 mg; pyridoxine, 2.4 mg; vitamin B_12_, 0.04 mg; pantothenic acid, 10 mg; niacin, 14 mg; biotin, 0.15 mg; folic acid, 0.2 mg

^b^ Provided the following per kilogram of diet: Fe (as ferrous sulfate), 120 mg; Cu (as copper sulfate), 80 mg; Mn (as manganese sulfate), 60 mg; Zn (as zinc sulfate), 120 mg; I (as potassium iodide), 0.3 mg; Se (as sodium selenite), 0.35 mg

#### Growth performance

The pigs were weighed individually after 12-h fasting in the morning of day 1 and day 22, and feed consumption per pen was recorded daily to calculated average daily feed intake (ADFI), average daily gain (ADG) and feed: gain (F/G).

### Exp. 2

#### Animals, diets and experimental design

A total of twenty healthy male pigs (Duroc × Landrace × Yorkshire), weaned at 24 days with an initial average body weight of 6.77 ± 0.10 kg, were randomly assigned into two dietary treatments (*n* = 10) based on their BW and litter, consisting of the basal diet (CON) or the basal diet supplemented with 400 mg/kg SDP. This selection of SDP level in Exp. 2 was based on the results of Exp. 1, which suggested that 400 mg/kg SDP resulted in lower F/G ratio and higher ADG (*P* < 0.05) than CON group. The basal and experimental diet were the same as described for Exp. 1, belonging to the same production batch. All pigs were housed in individual metabolism cages (0.7 m × 1.5 m) in a temperature-controlled conditions (26 ± 2 °C) with free access to food and water. The experimental period lasted for 21 days, and experimental procedures used were similar to those previously described for Exp. 1. Individual pig body weight was recorded after 12-hour fasting in the morning of day 1 and 22, and feed consumption was recorded daily. ADFI, ADG and F/G were calculated. The pigs’ diarrhea was observed at the same time of each morning throughout the experimental period as previously described [17]. The fecal consistency of each pig was assessed with a score from 0 to 3 (0 = normal feces, 1 = pasty feces, 2 = semiliquid feces, and 3 = liquid feces). The occurrence of diarrhea was defined as daily fecal consistency score of ≥2. Diarrhea rate per day in each treatment group was expressed as follows: diarrhea rate (%) = (number of pigs with diarrhea/total number of pigs) × 100, and the cumulative rate of diarrhea was calculated.

#### Sample collection and preparation

At the end of the experiment, after 12-hour fasting, eight pigs with weight close to the average level of each group were selected for bleeding via the anterior vena cava. Blood samples were centrifuged at 3000×*g* for 15 min at 4 °C to collect serum. After blood collection, the same pigs were then euthanized by intravenous injection of pentobarbital sodium. The gastrointestinal tract was immediately removed and divided into duodenum, jejunum, ileum and cecum. Approximately 2 cm segments of the middle of duodenum, jejunum and ileum were isolated, washed and fixed in 4% paraformaldehyde for morphological analysis. The mucosa samples of small intestine were collected by scraping the intestinal wall with glass microscope slides, frozen in liquid nitrogen and stored at − 80 °C until analysis. Finally, caecal digesta were snap frozen and stored at − 80 °C for microbial population measurements.

#### Serum parameters analysis

Serum antioxidant parameters including glutathione peroxidase (GSH-Px) activity, superoxide dismutase (SOD) activity, catalase (CAT) activity, total antioxidant capacity (T-AOC), and malondialdehyde (MDA) concentration were determined by the commercial kits (Nanjing Jiancheng Institute of Bioengineering, Nanjing, China) according to the manufacturer’s instructions. The level of interleukin (IL)-6, IL-10, IL-1β, tumour necrosis factor (TNF)-α, *D*-lactate and immunoglobulin (IgA, IgG and IgM) and diamine oxidase (DAO) activity were measured using commercially porcine-specific ELISA kits (Beijing Winter Song Boye Biotechnology Co. Ltd., Beijing, China).

#### Intestinal morphology

The fixed intestinal segments were dehydrated, and then embedded in paraffin, and cut into 5-μm thick sections. The sections were deparaffinized, rehydrated and stained with hematoxylin and eosin. At least four images per section and five sections from each pig were obtained. The villus height and crypt depth were determined on the images using an Olympus CK 40 microscope (Olympus Optical Company, Tokyo, Japan), and villi-crypt ratio (VCR) was calculated.

#### Small intestine biochemical analysis

The frozen small intestinal mucosa samples (approximately 0.5 g) were weighed and homogenized in ice-cold physiological saline solution (1:9, weight/volume), and then were centrifuged at 3500×*g* for 15 min at 4 °C to collect the supernatant. The secreted immunoglobulin A (sIgA) content in each section of small intestine was determined by the porcine ELISA assay kit (mlbio Biotech, Shanghai, China), and the results were expressed as mg sIgA/g intestinal protein. The activities of disaccharidase (sucrase, lactase and maltase) in jejunal mucosa were analysed using commercial assay kits (Nanjing Jiancheng Bioengineering Institute), and expressed as on a per milligram protein basis. The protein concentration was determined by Pierce BCA Protein Assay kit (Thermo Scientific, Waltham, MA, USA).

#### Quantitative real-time PCR (qRT-PCR) analysis

Total RNA was extracted from the small intestinal mucosa using TRIzol reagent (Sigma-Aldrich, Saint Louis, MO, USA) in accordance with the manufacturer’s instructions. The concentration and integrity of RNA was determined by nucleic-acid/protein analyzer (Beckman Coulter DU800, Beckman Coulter Inc., Fullerton, CA, USA) and 1% agarose gel electrophoresis, respectively. Reverse transcription reactions were conducted using iScript™ cDNA Synthesis Kit (Bio-Rad, Hercules, CA, USA). qRT-PCR was performed on the CFX96 RT-PCR Detection System (Bio-Rad) as described previously [17]. The relative mRNA expression of target genes was calculated and normalized with β-actin reference using the method of 2^−ΔΔCT^ [18]. The used primer sequences are shown in Table 2.

**Table 2:** Nucleotide sequences of primers used to measure targeted genes (Exp. 2)

Gene symbols	Nucleotide sequence of primers (5´→3´)	Product length, bp	Accession No.
β-actin	F: TCTGGCACCACACCTTCT R: TGATCTGGGTCATCTTCTCAC	78	XM_021086047.1
*TLR2*	F: ACGTATCCATCAATGAACACTGC R: AAGGGTGCAGTCATCAAACTC	146	NM_213761.1
*TLR4*	F: TCAGTTCTCACCTTCCTCCTG R: GTTCATTCCTCACCCAGTCTTC	121	GQ503242.1
*TLR6*	F: TCTCATGGCACAGCGAACTT R: ACATCATCCTCTTCAGCGACT	123	NM_213760.2
*MyD88*	F: GGTGCCAGGCAGGACATC R: GGCAGCTGGAACAGACCAA	68	NM_001099923.1
*IL-6*	F: GACAAAGCCACCACCCCTAA R: CTCGTTCTGTGACTGCAGCTTATC	69	M80258.1
*IL-1β*	F: TCTGCCTGTACCCCAACTG R: CCAGGAAGACGGGCTTTTG	63	NM214055.1
*TNF-α*	F: CGTGAAGCTGAAAGACAACCAG R: GATGGTGTGAGTGAGGAAAACG	121	EU682384.1
*IL-10*	F: GACCAGATGGGCGACTTGTT R: TGCCTTCGGCATTACGTCTT	120	NM_214041.1
*ZO-1*	F: GAGGATGGTCACACCGTGGT R: GGAGGATGCTGTTGTCTCGG	169	XM_021098896.1
Claudin-1	F: TCAATACAGGAGGGAAGCCAT R: ATATTTAAGGACCGCCCTCTCC	91	NM_001244539.1
Occludin	F: CAGGTGCACCCTCCAGATTG R: TGGACTTTCAAGAGGCCTGG	111	NM_001163647.2

*TLR* toll like receptor, MyD88 myeloid differentiation factor 88, *IL-6* interleukin-6, *IL-1β* interleukin-1β, *TNF-α* tumor necrosis factor α, *IL-10* interleukin-10, *TGF-β1* transforming growth factor-β1, *ZO-1* zonula occludens-1

#### Immunoblotting analysis

Immunoblotting analysis was performed as previously described [19]. Briefly, protein extracts from jejunal mucosa were separated by 10% SDS-PAGE and then transferred to PVDF membrane. Membranes were blocked with 5% non-fat dry milk in TBST for 1 h and overnight incubated at 4 °C with the primary antibodies against zonula occludens-1 (ZO-1), claudin-1, occludin, and β-actin (Proteintech, Chicago, IL, USA), followed by incubation with HRP-linked secondary antibody anti-rabbit IgG or anti-mouse IgG (Cell Signaling Technology, Danvers, USA) in TBST for 1 h at room temperature. Finally, membranes were visualized using Bio-Rad ChemiDoc™ imaging system (Bio-Rad). Band density of target protein was quantified after normalization to β-actin.

#### Quantification of microbial population

Bacterial DNA was extracted from caecal digesta using the Stool DNA kit (Omega Bio-Tek, Norcross, GA, USA) in accordance with the manufacturer’s instruction. The microbial qRT-PCR was analysis on the CFX-96 RT-PCR Detection System (Bio-Rad) as described previously [20]. Briefly, the number of total bacteria was determined using SYBR Premix Ex Taq reagents (TaKaRa Biotechnology, Dalian, China), and the PCR system was composed of 12.5 μL SYBR Premix Ex Taq (2×), 1 μL of forward and 1 μL of reverse primers (100 nmol/L), 9.5 μL ddH_2_O and 1 μL cDNA. Cycling conditions were as follows: 95 °C for 30 s, followed forty cycles of denaturation at 95 °C for 5 s, annealing at 60 °C for 30 s and extension at 72 °C for 60 s. The number of *Lactobacillus*, *Bacillus*, *E. coli* and *Bifidobacterium* were measured using PrimerScript™ PCR kit (TaKaRa), and the PCR system was composed of 8 μL RealMasterMix (2.5×), 1 μL of forward and 1 μL of reverse primers (100 nmol/L), 1 μL 20× probe enhancer solution, 0.3 μL probe (100 nmol/L), 1 μL DNA and 7.7 μL ddH_2_O. Cycling conditions were as follows: 95 °C for 2 min, followed fifty cycles of denaturation at 95 °C for 15 s, annealing at 60 °C for 30 s and extension at 72 °C for 50 s. For the quantification of bacteria in test samples, specific standard curves were generated by constructing standard plasmids as presented by Chen et al. [21]. The bacterial copies were transformed (log_10_) before statistical analysis. The used primers and probes were listed in Table 3.

**Table 3:** Primer and probe sequences used for real-time PCR

Items	Primer and probe sequence (5´→3´)	Product length, bp
*Total bacteria*	F: ACTCCTACGGGAGGCAGCAG R: ATTACCGCGGCTGCTGG	200
*Lactobacillus*	F: GAGGCAGCAGTAGGGAATCTTC R: CAACAGTTACTCTGACACCCGTTCTTC	126
	P: AAGAAGGGTTTCGGCTCGTAAAACTCTGTT	
*Bifidobacterium*	F: CGCGTCCGGTGTGAAAG R: CTTCCCGATATCTACACATTCCA	121
	P: ATTCCACCGTTACACCGGGAA	
*Bacillus*	F: GCAACGAGCGCAACCCTTGA R: TCATCCCCACCTTCCTCCGGT	92
	P: CGGTTTGTCACCGGCAGTCACCT	
*Escherichia coli*	F: CATGCCGCGTGTATGAAGAA R: CGGGTAACGTCAATGAGCAAA	96
	P: AGGTATTAACTTTACTCCCTTCCTC	

#### Determination of caecal short-chain fatty acid (SCFAs) concentrations

The concentration of main SCFAs in caecal digesta was determined by gas chromatography as previously described [22]. Digesta samples were weighed and then centrifuged to obtain the supernatants after adding distilled water. The supernatant (1 mL) was mixed with 0.2 mL metaphosphoric acid, followed incubation at 4 °C for 30 min. The mixture was centrifuged at 12,000 × *g* for 10 min at 4 °C and 1 μL of the supernatant was measured by injection into the gas chromatograph system (GC-2014, Shimadzu Corporation, Kyoto, Japan).

### Statistical analysis

The experimental design of Exp.1 was a completely randomized block design based on initial BW, sex and litter. For the growth performance in Exp. 1, data were analyzed using the GLM procedure of SAS (SAS Institute Inc., Cary, NC, USA), with pen as the experimental unit. Significant differences among groups were determined by Duncan’s multiple range test. Mortality rate of pigs were analyzed using chi-square test. For all other variables in Exp. 2, data were analyzed by using an unpaired two-tailed Student’s *t*-test, with the selected pig as the experimental unit. Results were presented as means and standard error (SE) except for the mortality rate of pigs as percentage. A significant difference was considered as *P*<0.05.

## Results

### Growth performance and diarrhea index (Exp. 1)

As shown in Table 4, there were no significant differences in ADFI between the pigs fed the CON diet and the SDP diets. However, dietary supplementation with 400 or 800 mg/kg SDP increased ADG (*P*<0.05) and decreased F/G ratio (*P*<0.05) compared with the CON group. No significant difference in growth performance between the pigs fed the CON diet and 200 mg/kg SDP-supplemented diet. Moreover, mortality rate of pigs was decreased numerically by SDP supplements (6.67% in CON, 3.33%, 1.67% and 1.67%, respectively, in 200, 400 and 800 mg SDP).

**Table 4:** Effects of dietary seaweed-derived polysaccharides (SDP) on growth performance of weaned pigs (Exp. 1)

Items	CON	Dietary SDP, mg/kg			*P*-value
		200	400	800	
Initial BW, kg	6.77 ± 0.09	6.75 ± 0.08	6.80 ± 0.09	6.87 ± 0.09	0.779
Final BW, kg	12.24 ± 0.18^b^	12.65 ± 0.26^b^	13.24 ± 0.13^a^	13.47 ± 0.17^a^	0.001
ADFI, g	386.1 ± 5.3	392.3 ± 13.0	409.4 ± 7.7	418.1 ± 13.7	0.150
ADG, g	260.5 ± 7.3^b^	281.1 ± 10.3^b^	306.7 ± 4.7^a^	314.2 ± 5.5^a^	< 0.001
F/G	1.49 ± 0.04^b^	1.47 ± 0.03^b^	1.34 ± 0.02^a^	1.36 ± 0.05^a^	0.004
Mortality, %	6.67	3.33	1.67	1.67	0.376

*ADFI* average daily feed intake; *ADG* average daily gain; *F/G* feed: gain

*n* = 6 for each group ^a,b^Mean values with unlike superscript letters were significantly different (*P*<0.05)

### Growth performance (Exp. 2)

As shown in Table 5, the SDP-supplemented pigs had greater ADG (*P*<0.05) and lower F/G ratio (*P*<0.05) compared to those in the CON group. No significant difference in ADFI was observed between the two groups. In addition, pigs fed the SDP diet had lower diarrhea rates (*P*<0.05) than those fed the CON diet during the overall period. There was no mortality in either group.

**Table 5:** Effects of dietary seaweed-derived polysaccharides (SDP) on growth performance and diarrhea rate of weaned pigs (Exp. 2)

Items	Dietary treatment		*P*-value
	CON	SDP	
Initial BW, kg	6.73 ± 0.11	6.81 ± 0.10	0.597
Final BW, kg	12.85 ± 0.09	13.52 ± 0.18	0.005
ADFI, g	415.7 ± 8.6	431.8 ± 7.6	0.185
ADG, g	291.6 ± 4.6	319.7 ± 4.8	0.001
F/G	1.43 ± 0.02	1.35 ± 0.02	0.025
Diarrhea rate, %	7.86 ± 0.56	4.51 ± 1.01	0.036

*ADFI* average daily feed intake; *ADG* average daily gain; *F/G* feed: gain

*n* = 8 for each group

### Antioxidant capacity (Exp. 2)

The serum antioxidant parameter results are shown in Table 6. Dietary SDP supplementation increased the activities of GSH-Px, SOD and CAT in serum (*P*<0.05). Meanwhile, pigs fed SDP diet tended to have a increased T-AOC activity and reduced MDA content in serum compared with those in the CON group (*P*<0.10).

**Table 6:** Effects of dietary seaweed-derived polysaccharides (SDP) on the serum antioxidant status of weaned pigs (Exp. 2)

Items	Dietary treatment		*P*-value
	CON	SDP	
GSH-Px, U/mL	352.45 ± 18.33	438.38 ± 22.07	0.024
SOD, U/mL	60.17 ± 3.64	77.80 ± 1.96	0.005
CAT, U/mL	11.83 ± 0.96	15.98 ± 0.85	0.006
T-AOC, U/mL	4.73 ± 0.16	5.23 ± 0.17	0.054
MDA, nmol/mL	5.05 ± 0.33	4.05 ± 0.27	0.064

*GSH-Px* glutathione peroxidase; *SOD* superoxide dismutase; *CAT* catalase; *T-AOC* total antioxidant capacity; *MDA* malondialdehyde

*n* = 8 for each group

### Immune indices (Exp. 2)

After dietary SDP inclusion, higher IL-6 and TNF-α concentrations in the serum were observed relative to CON group (*P*<0.05). Meanwhile, pigs on the SDP group tended to have a increased IgG content (*P* = 0.090) compared to those on the CON group. Dietary SDP supplementation increased mucosal sIgA content in all of the selected intestinal segments (*P*<0.05) (Table 7).

**Table 7:** Effects of dietary seaweed-derived polysaccharides (SDP) on the immune responses of weaned pigs (Exp. 2)

Items	Dietary treatment		*P*-value
	CON	SDP	
Serum			
IgG, g/L	19.63 ± 0.86	21.98 ± 0.78	0.090
IgA, g/L	1.25 ± 0.09	1.30 ± 0.05	0.653
IgM, g/L	2.34 ± 0.04	2.39 ± 0.06	0.540
IL-6, pg/mL	127.14 ± 4.98	159.31 ± 7.35	0.003
IL-10, pg/mL	14.65 ± 2.47	17.36 ± 1.23	0.342
IL-1β, pg/mL	31.68 ± 3.32	33.48 ± 2.28	0.662
TNF-α, pg/mL	57.61 ± 3.90	70.76 ± 4.56	0.046
Mucosal sIgA, mg/g protein			
Duodenum	3.35 ± 0.66	5.56 ± 0.74	0.043
Jejunum	4.78 ± 0.50	8.14 ± 0.53	< 0.001
Ileum	5.68 ± 0.46	9.04 ± 0.61	0.001

*IgG* immunoglobulin G; *IgA* immunoglobulin A; *IgM* immunoglobulin M; *IL-6* interleukin-6; *IL-10* interleukin-10; *IL-1β* interleukin-1β; *TNF-α* tumour necrosis factor-α; *sIgA* secretory immunoglobulin A

*n* = 8 for each group

### Intestinal morphology (Exp. 2)

The data of intestinal morphology were shown in Table 8. In the duodenum, dietary supplementation of SDP significantly increased villous height and the VCR index and decreased crypt depth compared with the control group (*P*<0.05). In the Jejunum, pigs fed the SDP diet had higher villus height and VCR index than those fed the CON diet (*P*<0.05). A similar histomorphologic change was observed in the ileum.

**Table 8:** Effects of dietary seaweed-derived polysaccharides (SDP) on intestinal morphology of weaned pigs (Exp. 2)

Items	Dietary treatment		*P*-value
	CON	SDP	
Villus height, μm			
Duodenum	212.78 ± 14.37	277.50 ± 11.06	0.003
Jejunum	197.18 ± 10.81	281.56 ± 11.99	< 0.001
Ileum	168.12 ± 4.89	243.74 ± 10.90	< 0.001
Crypt depth, μm			
Duodenum	105.24 ± 3.49	91.47 ± 2.83	0.008
Jejunum	96.75 ± 4.91	87.59 ± 2.25	0.121
Ileum	76.91 ± 3.17	67.88 ± 3.05	0.059
VCR			
Duodenum	2.05 ± 0.18	3.06 ± 0.17	0.001
Jejunum	2.07 ± 0.15	3.24 ± 0.18	< 0.001
Ileum	2.21 ± 0.09	3.67 ± 0.28	0.001

*VCR* villus height: crypt depth ratio

*n* = 8 for each group

### The activities of disaccharidase in jejunal mucosa (Exp. 2)

As shown in Table 9, pigs fed the SDP diet had markedly increased the activities of lactase and maltase in jejunal mucosa compared with those fed the CON diet (*P*<0.05). However, no significant difference in sucrase activity was observed between the two groups.

**Table 9:** Effects of dietary seaweed-derived polysaccharides (SDP) on the activities of disaccharidases in jejunal mucosa of weaned pigs (Exp. 2)

Items	Dietary treatment		*P*-value
	CON	SDP	
Lactase, U/mg protein	104.97 ± 3.28	118.40 ± 7.58	0.008
Sucrase, U/mg protein	75.60 ± 4.40	91.05 ± 8.33	0.199
Maltase, U/mg protein	144.41 ± 9.48	240.46 ± 11.19	0.001

*n* = 8 for each group

### Serum diamine oxidase activity and *D*-lactate content (Exp. 2)

**Fig. 1:**
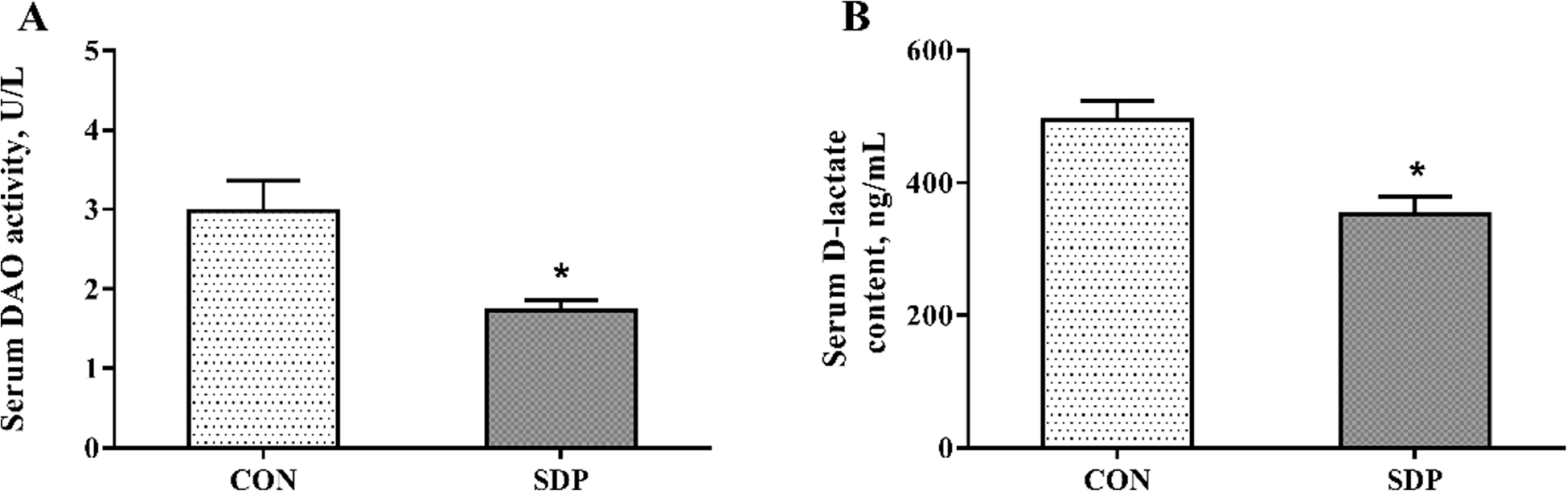
Effects of dietary seaweed-derived polysaccharides (SDP) on serum diamine oxidase (DAO) activity and *D*-lactate content in weaned pigs. **a** serum DAO activity; **b** serum *D*-lactate content. Data are expressed as means ± SEM, *n* = 8, * *P* <0.05

As shown in Fig. 1, dietary supplementation with SDP significantly decreased the DAO activity and *D*-lactate content in serum compared with the CON group (*P*<0.05).

### Intestinal inflammatory response and barrier functions related genes expression (Exp. 2)

**Fig. 2:**
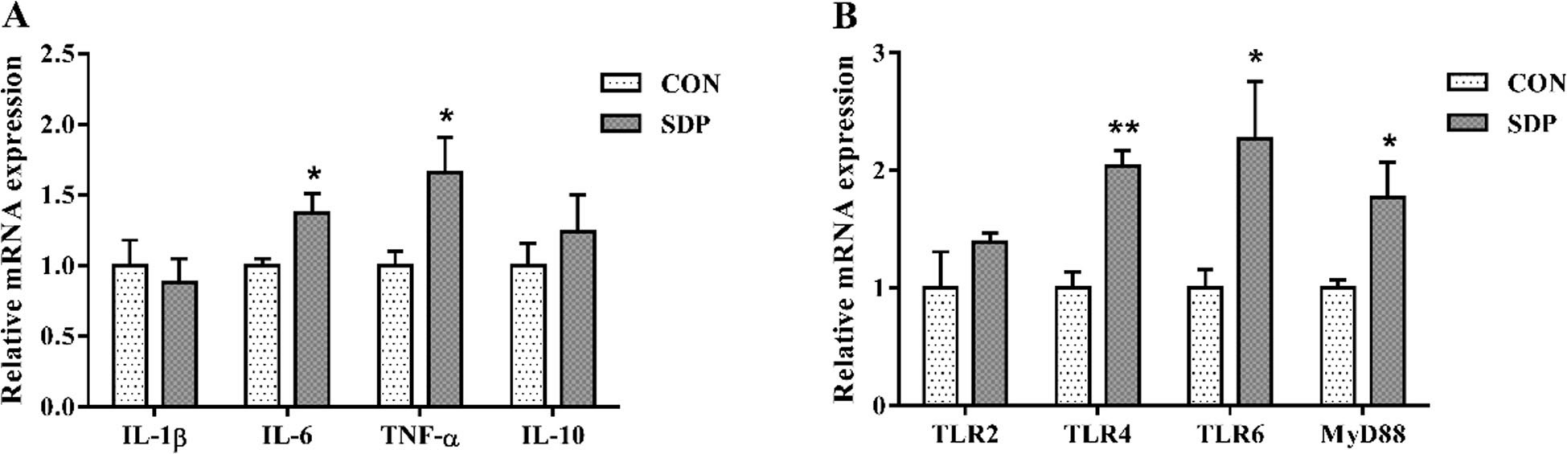
Effects of dietary seaweed-derived polysaccharides (SDP) on mRNA expression of immune related genes in jejunal mucosa of weaned pigs. **a** interleukin (IL)-1β, IL-6, tumour necrosis factor (TNF)-α and IL-10; **b** toll-like receptor (TLR)-2, TLR-4, TLR-6 and myeloid differentiation factor 88 (MyD88). Data are expressed as means ± SEM, *n* = 8, * *P* <0.05, **P <0.01

**Fig. 3:**
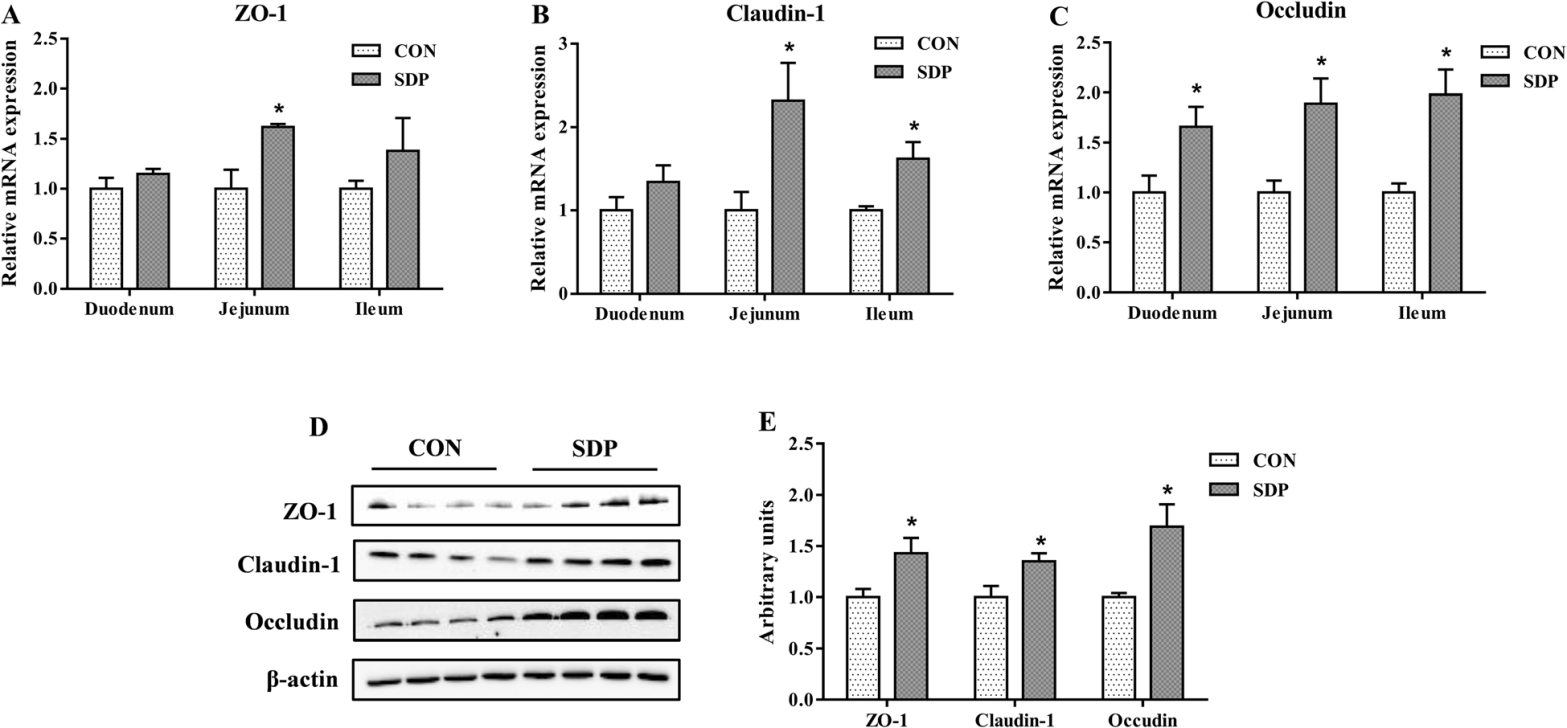
Effects of dietary seaweed-derived polysaccharides (SDP) on expression levels of genes related to intestinal barrier functions in weaned pigs. **a-c** mRNA expression of zonula occludens protein-1 (ZO-1) (**a**), claudin-1(**b**) and occludin (**c**) in small intestine; **d**-**e** representative images of immunoblotting (**d**) and protein expression of ZO-1, claudin-1 and occludin (**e**) in jejunal mucosa. Data are expressed as means ± SEM, *n* = 8, * *P* <0.05

As shown in Fig. 2, dietary supplementation of SDP increased the mRNA abundance of *IL-6*, *TNF-α*, toll like receptor *(TLR)* 4, *TLR6* and myeloid differentiation factor 88 *(MyD88)* in the jejunum mucosa of pigs (*P*<0.05). In addition, the expression levels of several critical genes related to intestinal barrier functions were shown in Fig. 3. Compared to the CON group, SDP-supplemented pigs had higher occludin mRNA expression level in the duodenum mucosa (*P*<0.05). Dietary SDP supplementation also elevated the mRNA expression levels of *ZO-1*, claudin-1 and occludin in the jejunum mucosa (*P*<0.05), and elevated the mRNA expression levels of claudin-1 and occludin in the ileum mucosa (*P*<0.05). Consistently, the protein expressions of ZO-1, claudin-1 and occludin in the jejunum mucosa were increased by SDP supplementation (*P*<0.05).

### Intestinal microbial populations and metabolites (Exp.2)

**Fig. 4:**
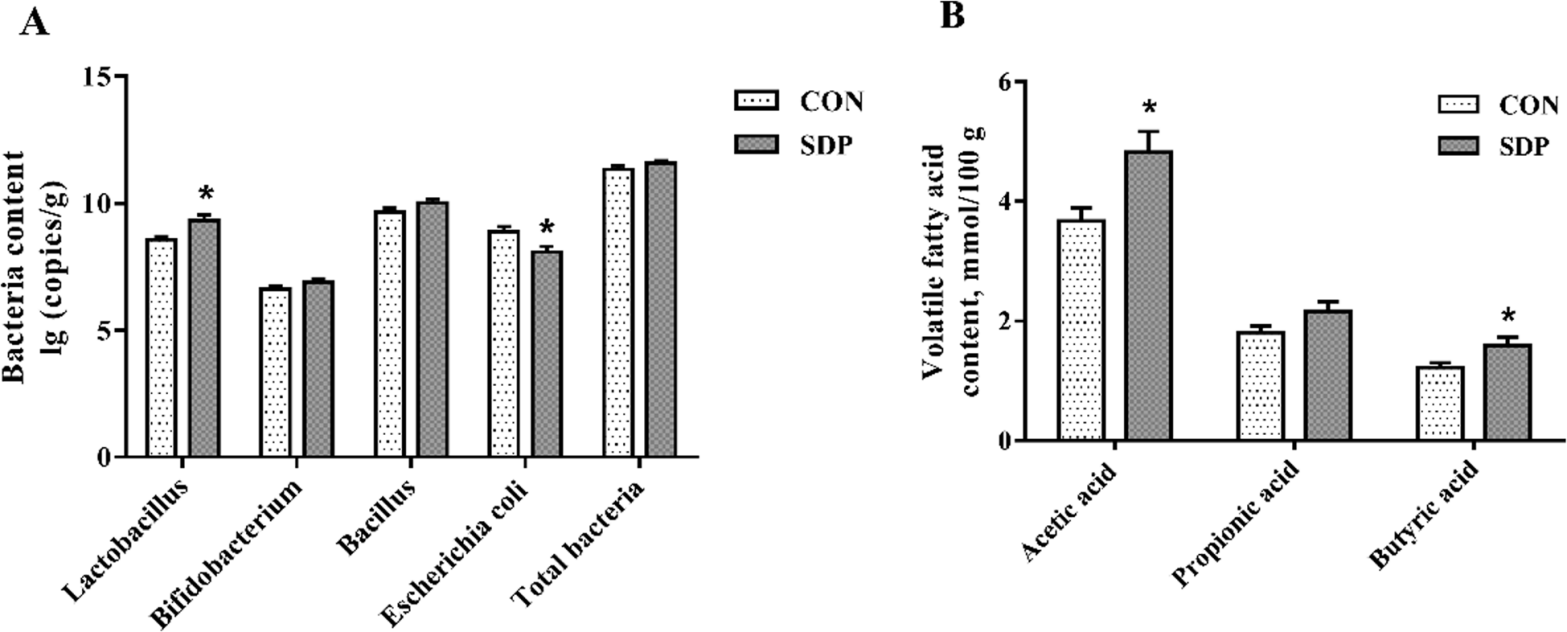
Effects of dietary seaweed-derived polysaccharides (SDP) on intestinal microbial population and metabolites in weaned pigs. **a** selected microbial population in the cecum. **b** volatile fatty acid concentration in the cecum. Data are expressed as means ± SEM, *n* = 8, * *P* <0.05

As shown in Fig. 4, dietary SDP supplementation significantly increased the *Lactobacillus* population and reduced the *Escherichia coli* population in the cecum of pigs (*P*<0.05). Moreover, compared to the CON group, SDP-supplemented pigs had higher acetic acid and butyric acid concentrations in the cecal digesta (*P*<0.05).

## Discussion

In recent years, natural bioactive polysaccharides have attracted extensive attention worldwide since their usefulness in regulating the gut health and metabolism [23]. Seaweeds are considered a potential source of useful metabolites and bioactive polysaccharide compounds, with wide variety of biological and physiological activities [24]. The results of the present study first showed that proper dietary *Enteromorpha* polysaccharides supplementation improved the growth performance in weaned pigs, which is similar to previous report in juvenile shrimp [16]. Another intriguing discovery in the present study is that the occurrence of diarrhea in pigs fed SDP-supplemented diet was significantly decreased. In order to clarify the mechanism of health-promoting effect of dietary SDP, the metabolic and intestinal responses of weaned pigs to the SDP diet were further investigated.

Oxidative stress, defined as the disequilibrium between the generation of reactive oxygen species (ROS) and the antioxidant network, is considered as an essential pathogenic factor in the development of gastrointestinal mucosal diseases [25]. Weaning is the most severe early-life stress for pigs, resulting in the damage of antioxidant system [26]. The complex enzymatic antioxidant defense system including GSH-Px, SOD and CAT are involved in protecting the organism from the damaging effects of ROS [27]. In detail, SOD plays a crucial role in scavenging superoxide radicals, whereas CAT is mainly responsible for eliminating organic hydroxyl radicals [28]. The current study showed that dietary SDP supplementation enhanced the activities of SOD, CAT and GSH-Px in serum, which was similar to the previous study in laying hens [6]. The improved antioxidant defense systems function of weaned pigs by dietary SDP might also contribute to the improvement in growth performance in the present study.

Previous study demonstrated that *Enteromorpha*-derived polysaccharides promoted immune response in juvenile shrimps by modulating hemolymph immune enzyme activities and immune-related gene expression [16]. Similarly, we here observed that a humoral immune response might be enhanced in SDP-supplemented pigs, as indicated by the increased serum concentrations of IL-6 and TNF-α. Furthermore, it has been demonstrated that sIgA acts as the first-line defense barrier in protecting the intestinal epithelium from enteric toxins and pathogens by agglutinating them and facilitating their clearance by peristaltic and mucociliary movements [29]. In the present study, SDP supplementation activated the intestinal mucosal immunity according to the higher sIgA content in small intestine. In agreement, the gene expression of *IL-6* and *TNF-α* were also increased in jejunal mucosa of SDP-supplemented pigs. The TLR4 signaling cascade has been demonstrated as a classic natural polysaccharide-regulated pathway [30]. In this study, feeding SDP diet significantly increased the mRNA expression levels of *TLR4*, *TLR6* and *MyD88* in jejunal mucosa, indicating that SDP may enhance the intestinal immune-response capacity after weaning via TLR4-MyD88 signaling.

The weakness in digestion and absorption of nutrients in intestine are responsible for the growth retardation and diarrhea observe in weaned pigs [17]. The high ratio of villus height to crypt depth in small intestine has been regarded as a marker of the improved nutrient digestion-absorption capacity [31]. Polysaccharides have been shown to promote intestinal epithelial growth and balance gut microflora in livestock [32]. In this study, the VCR index in duodenum, jejunum and ileum of weaned pigs were increased by SDP supplementation. Consistent with the positive effect of SDP on intestinal morphology, feeding SDP diet increased the activities of lactase and maltase in the jejunum. This reflects an improvement in nutrient digestibility and may be important factors to promote the postweaning growth. Similarly, previous study suggested that dietary supplementation with seaweed extracts containing laminarin and fucoidan increased nutrient digestibility in piglets during the weaning period [33]. Furthermore, the intestinal epithelial barrier disruption, characterized by increased intestinal permeability, induces a penetration of pathogens, toxins, and antigens, negatively affecting absorption of nutrients [34]. The selectively permeable barrier is achieved by typical structural proteins of epithelial tight junction, including ZO-1, claudin-1 and occludin [34, 35]. We found that SDP supplementation promoted jejunal structural integrity, as shown by the increased mRNA and protein expression of ZO-1, claudin-1 and occludin in jejunal mucosa of pigs. The result is consistent with the measurements of the intestinal permeability by using the blood indices. DAO and *D*-lactate levels in the blood were usually used as useful biomarkers for monitoring the integrity of intestinal barrier [36, 37]. In the present study, feeding SDP diet significantly decreased the serum DAO activity and *D*-lactate concentration in pigs. Taken together, these results suggested that SDP supplementation could improve the intestinal barrier function. In addition, it is well known that the signaling extent of TLR4-MyD88 pathway has closely associated with the intestinal barrier [38]. The improved integrity of intestinal barrier indicates there was decreasing risk of inflammatory bowel disease in the current study.

The intestinal microbiota, which depends mostly on non-digestible fibers and polysaccharides as energy sources, plays a vital role in many aspects of host physiology [39]. Previous studies indicated that marine polysaccharides can be efficiently catabolized and utilized by beneficial bacteria in caeco-colon [40]. In this regard, insights into the effects of SDP on intestinal ecology are of vital importance for understanding the beneficial effects of the polysaccharides. We hypothesized that *Enteromorpha* polysaccharides may exert beneficial effects on intestinal barrier function partially through regulating gut microbiota composition and metabolites. As expected, feeding SDP diet elevated *Lactobacillus* population, but reduced *Escherichia coli* population in the cecum of pigs. The result is similar to previous studies that the addition of fucoidan increased the lactobacilli population in the caecum of newly weaned pigs challenged with *Salmonella* Typhimurium [41], and seaweed extract containing laminarin and fucoidan inhibited the growth of *Escherichia coli* in pigs after weaning [42, 43]. Pigs offered diets containing fucoidan had increased lactobacilli spp. in colonic digesta [44]. Furthermore, diets could influence gut microbiota community and then impact different SCFAs production [45]. The SCFAs produced by intestinal flora metabolism, mainly acetic acid, propionic acid and butyric acid, also play an essential role in improving intestinal epithelial barrier function. Butyrate acid serves as the sources of fuel for intestinal epithelial cells, which can promote the function of intestinal mucosal barrier and mitigate colitis [46, 47]. The acetic and propionic acids are reported to enhance the tight junction barrier integrity in rat colons and cultured intestinal cells [48]. Previous study suggested that *Enteromorpha prolifera* polysaccharides were readily fermented by human gut microbiota *in vitro* and produced various SCFAs. Besides, *Enteromorpha prolifera* polysaccharides stimulated the population growth of intestinal *Lactobacillus* during fermentation process [49]. In the present study, SDP supplementation significantly increased the concentrations of acetic acid and butyric acid and *Lactobacillus* population in the cecal digesta, which provides a potential mechanism for SDP-improved intestinal barrier functions. Taken together, these results suggested a beneficial effect of SDP on intestinal microbial ecology and health.

## Conclusions

In conclusion, our results demonstrated a potential beneficial role of SDP in improving the growth performance and intestinal health in weaned pigs, possibly via mechanisms associated with suppressing oxidative stress, improving intestinal morphology and barrier functions, and changes of the immune status and microbial fermentation. These findings indicate the potential application of SDP as a safe and effective nutritional intervention strategy to maintain gut health in neonatal mammals.

## Data Availability

The data produced or analyzed during the current study are available from the corresponding author by reasonable request.

## Abbreviations

BW: Body weight
CAT: Catalase
DAO: Diamine oxidase
GSH-Px: Glutathione peroxidase
IL: Interleukin
MDA: Malondialdehyde
MyD88: Myeloid differentiation factor 88
SCFA: Short-chain fatty acid
SDP: Seaweed-derived polysaccharides
sIgA: Secreted immunoglobulin A
SOD: Superoxide dismutase
T-AOC: Total antioxidant capacity
TLR: Toll like receptor
TNF-α: Tumour necrosis factor-α
VCR: Villi-crypt ratio
ZO-1: Zonula occludens-1
